# Bioflavonoid exerts analgesic and anti-inflammatory effects via transient receptor potential 1 channel in a rat model

**DOI:** 10.1055/s-0042-1755321

**Published:** 2022-11-09

**Authors:** Mohammad Mahdi Zarei, Zohreh Abdolmaleki, Siamak Shahidi

**Affiliations:** 1 Islamic Azad University, Department of Pharmacology, Karaj, Iran.; 2 University of Medical Sciences, School of Medicine, Department of Physiology, Hamadan, Iran.; 3 Hamadan University of Medical Sciences, Neurophysiology Research Center, Hamadan, Iran.

**Keywords:** Analgesia, TRPA1 Cation Channel, Rats, Flavonoids, Analgesia, Canal de cátion TRPA1, Ratos, Flavonoides

## Abstract

**Background** Pain is an uncomfortable sensation in the body. Kaempferol is a flavonoid with antinociceptive effects. Transient receptor potential (TRP) channels have been characterized in the sensory system.

**Objective** This study evaluated the central antinociceptive effect of Kaempferol and possible mechanisms of action of transient receptor potential cation channel subfamily V member 1 (TRPV1).

**Methods** Capsaicin as a TRPV agonist (5 μg/μL, intracerebroventricular [ICV]) and capsazepine as its antagonist (10 μg/μL, icv) were used to test the analgesic effect of kaempferol (1.5 mg, ICV). Morphine (10 μg, ICV) was used as a positive control. The other groups were treated with a combination of kaempferol and capsaicin, kaempferol and capsazepine, and capsaicin and capsazepine. The cannula was implanted in the cerebroventricular area. The tail-flick, acetic acid, and formalin tests were used to assess analgesic activity. For evaluation of antiinflammatory effect, the formalin-induced rat paw edema was used.

**Results** Kaempferol significantly decreased pain in the acute pain models, including the tail-flick and the first phase of the formalin test. In the late phase of the formalin test, as a valid model of nociception, capsazepine inhibited the antinociceptive effect of kaempferol.

**Conclusions** Kaempferol has an analgesic effect in the acute pain model and can affect inflammatory pain. Also, the TRPV1 channel plays a role in the antinociceptive activity of kaempferol.

## Resumo

**Antecedentes** A dor é uma sensação desconfortável no corpo. Kaempferol é um flavonoide com efeitos antinociceptivos. Canais receptores de potencial transitório têm sido caracterizados no sistema sensorial.

**Objetivo** Este estudo avaliou o efeito antinociceptivo central do kaempferol e os possíveis mecanismos de ação do TRPV1.

**Métodos** Capsaicina como agonista de TRPV (5 μg/μL, intracerebroventricular [ICV]) e capsazepina como seu antagonista (10 μg/μL, icv) foram usados para testar o efeito analgésico do kaempferol (1,5 mg, ICV). A morfina (10 μg, ICV) foi usada como controle positivo. Os outros grupos foram tratados com uma combinação de kaempferol e capsaicina, kaempferol e capsazepina e capsaicina e capsazepina. A cânula foi implantada na área cerebroventricular. Os testes de movimento de cauda, ácido acético e formalina foram usados para avaliar a atividade analgésica. Para avaliação do efeito anti-inflamatório, foi utilizado o edema de pata de rato induzido por formalina.

**Resultados** Kaempferol diminuiu significativamente a dor nos modelos de dor aguda, incluindo o movimento da cauda e a primeira fase do teste de formalina. Na fase tardia do teste da formalina, como modelo válido de nocicepção, a capsazepina inibiu o efeito antinociceptivo do kaempferol.

**Conclusões** Kaempferol tem efeito analgésico no modelo de dor aguda e pode afetar a dor inflamatória. Além disso, o canal TRPV1 desempenha um papel na atividade antinociceptiva do kaempferol.

## INTRODUCTION

Pain is an unpleasant feeling that has always been a serious challenge in medicine, as it has an important protective role in avoiding treating genuine or potential tissue damage. 1 It has been reported that patients sustained chronic and high-impact chronic pain (20.4% and 8.0%, respectively) in the United States. 2 Nowadays, several substances, including opioids and non-steroidal antiinflammatory drugs are used to treat pain. However, opioids cause dependency and tolerance 3 ^,^ and non-steroidal antiinflammatory drugs cause gastrointestinal disorders. 1

Medicinal plants can be a source for finding newer combinations of drugs. Because of establishing a biological balance of active ingredients in medicinal plants and no accumulation in the body, these plants have fewer side effects or no significant side effects compared with chemical pain relievers. 4 5 It is essential to find new drugs or better understand the pharmaceutical effects of medicinal plants.

Kaempferol is a dietary bioflavonoid found in a variety of plants. 6 It successfully crosses the blood-brain barrier and causes changes in the brain. 6 Kaempferol is a secondary metabolite with anticarcinogenic, 7 antiamnesia, 8 antioxidant, antiinflammatory, 6 and antinociceptive effects in diabetic rats. 9 10

The protective effect of flavonoids against inflammation through the regulation of the transient receptor potential cation channel subfamily V member 1 (TRPV1) has been reported. 11 The TRPV1 is a member of the TRP channels. 11 The TRPV1 receptors are activated by capsaicin, noxious heat, and acid. 12 TRPV1 receptors are involved in somatic and visceral peripheral inflammation in the spinal cord and brainstem centers. 13

The activation of TRPV1 receptors in the peripheral nerve endings produces a calcium and sodium influx that ultimately results in nociceptive sensitization (pronociceptive effect). 14 15 However, the use of capsaicin in the central nerve endings induces an increase in Ca ^2+^ in the spinal cord and induces an antinociceptive effect. 13

The interaction between the kaempferol and TRPV1 in the central nervous system is not well defined. The aim of the present study was to evaluate and compare the effects of kaempferol and capsaicin for a better understanding of the mechanism of action of kaempferol. This study investigated the effects of the interaction between kaempferol and both the agonist and antagonist of TRPV1.

## METHODS

### Drugs

Kaempferol, as a natural flavanol, capsaicin, as a vanilloid type 1 receptor agonist, capsazepine, as a synthetic antagonist of capsaicin, morphine sulfate, ketamine, xylazine, and formalin were purchased from Sigma-Aldrich (St. Louis, MO, USA). Kaempferol, capsaicin, and capsazepine were dissolved in 99.9% dimethyl sulfoxide (DMSO), whereas the other compounds were suspended in 0.9% physiological saline.

### Animals

Fifty-six male Wistar rats (weighing 200–250 g; Pasteur Institute, Iran) were used in this study. The animals had free access to water and food and were kept under a 12:12 hour light-dark cycle (humidity, 50 ± 5%; temperature, 22 ± 2°C). The experiments were performed in the light period (10:00–16:00). The examiner was blinded to the treatments, and the rats were classified randomly into different groups. Animal experiments were conducted following the guidelines for the care and use of laboratory animals. The study protocol was approved by the research ethics committee of Islamic Azad University, Karaj Branch (Iran), and the department of pharmacology, Faculty of Veterinary Medicine (IR.IAU.K.REC.1397.035).

### Experimental groups

Six rats were considered for each experimental group, including control (vehicle), positive control, morphine (10 µg/rat), kaempferol (1.5 mg /rat), 16 capsaicin (0.005 µM/rat), capsazepine, (0.01 µM/rat), capsaicin plus kaempferol, and capsazepine plus kaempferol, and capsaicin plus capsazepine.

In the capsazepine and capsaicin group, the ED50 of the agonist was selected first. The capsazepine was injected into the rats for blocking the vanilloid type 1 receptors and then, capsaicin (ED50) was injected along with capsazepine. Capsaicin competes with capsazepine and exerts its effect.

Because the intracerebroventricular (ICV) injection is a safe and well-tolerated route of administration for long-term treatment, 17 18 the compounds were administered intracerebroventricularly.

### Intracerebroventricular cannulation implantation

First, the rats were intraperitoneally anesthetized using a mixture of ketamine (80 mg/kg) and xylazine (10 mg/kg). Then, a stainless-steel cannula (21-gauge; 12 mm) was placed in the right lateral ventricle for ICV injection through stereotaxic surgery. According to the literature, the stereotaxic coordinates were as follows: 1.5 mm lateral, 0.8 mm posterior, and 4.0 mm ventral to the bregma. 19

The guide cannula was fixed to the skull using two stainless steel small screws anchored to the skull and dental acrylic cement, and then, sealed using a stainless steel wire for inhibiting occlusion. The rats were excluded from the experimental groups if the cannula moved during the experiments.

After 7 days of recovery, the rats were separately housed before the experiments. The drug solution was added to an injection cannula (29-gauge 15 mm), which was attached to a Hamilton syringe (10-μl) by a PE-20 catheter, and then, added to a guide cannula extending 1 mm beyond the tip of the guide cannula. To conduct each task, saline (10 μl), kaempferol (1.5 µg), capsaicin (5 nM), capsazepine (10 nM), and morphine (10 µg) were separately delivered over 20 minutes.

### Abdominal writhing test

The animals were transferred to a small plastic box for habituation 30 minutes before the experiments. The intended compound was dissolved in the vehicle, and then it (10 μL) was injected slowly into the ICV system using a Hamilton syringe equipped with an injection cannula and a catheter. After 10 minutes, acetic acid with a density of 6% was intraperitoneally injected at 10 ml/kg, and, immediately, the number of abdominal contractions was counted for 30 minutes with both legs of the animals stretched.

### Tail-flick test

The antinociceptive response against thermal stimulus was assessed with the tail-flick test. The rats were restrictively held with the tail on a slot (adjustable width) with a groove to ensure accurate placement in the tail-flick apparatus for radiant thermal stimulation of the dorsal surface of the rear. The intensity of the thermal stimulus was adjusted to cause the animal to flick its tail within 2 to 4 seconds as the baseline of the tail-flick latency. The gap between the start of heat exposure and tail withdrawal was calculated to determine the tail-flick latency. The tail-flick latency was measured at zero, 30, 60, 90, and 120 minutes after the central administration of the compounds (as described above). The cut-off time was set at 10 seconds to minimize tissue damage.

### Formalin test

The formalin test was used for the evaluation of acute and inflammatory pain and was done after the tail-flick test. The rats were injected with 50 μl of 2.5% formalin (formaldehyde solution 37% [w/w] diluted in saline) that was injected into the sub plantar space of the right hind paw 10 minutes after central treatment with drugs. The control animals received saline before formalin injection. Then, the animals were placed in the special plexiglass box (30 × 30 × 30 cm). A mirror was placed at a 45° angle beneath the box. This mirror allows a clearer observation of the animals' behaviors. The time spent on licking, biting, and shaking behaviors was measured from 0 to 5 minutes (the early phase) and 5 to 45 minutes (the late period) after formalin injection. The sham animals received 0.9% saline injected into the paw to make sure about the formalin-induced peripheral sensitization.

### Statistical analysis

Data are presented as the mean ± standard error of the mean (SEM) in this study. Data were analyzed using the SPSS Statistics for Windows, version 16.0 software (SPSS Inc., Chicago, IL, USA) and the Kolmogorov-Smirnov test followed by repeated measures analysis of variance (ANOVA) and Tukey posthoc test. The significant difference was set at a *p* -value< 0.05.

## RESULTS

### Results of the abdominal writhing test

**Figure 1:**
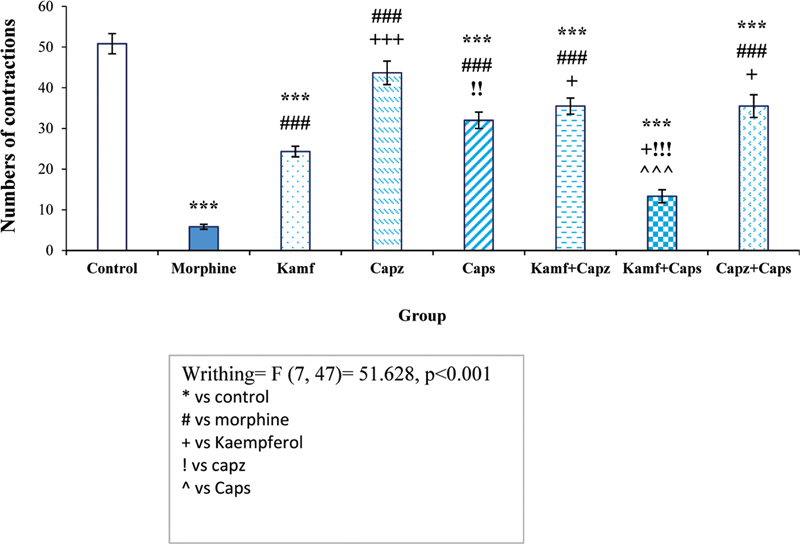
Comparison of the number of abdominal contractions in the writhing test. *** ( *p* < 0.001) compared with the control group; ### ( *p* < 0.001) compared with the morphine group; +++ ( *p* < 0.001) compared with the kaempferol group; !!! ( *p* < 0.001) and !! ( *p* < 0.01) compared with the capsazepine group; ^^^ ( *p* < 0.001) compared with the capsaicin group. Abbreviations: KM, kaempferol; Capz, capsazepine; Caps, capsaicin. Each column represents the mean ± SEM. *** ( *p* < 0.001), ** ( *p* < 0.01), and * ( *p* < 0.05) compared with the control group.

According to Figure 1 , there was a significant difference between the experimental groups (F [7, 47] = 51.628, *p* < 0.001). The statistical analysis revealed that kaempferol led to a significant decrease in abdominal pain compared with the control group ( *p* < 0.001, respectively), and morphine also significantly reduced the abdominal pain score compared with the other group ( *p* < 0.001).

The Tukey posthoc test showed that there was a significant difference between the capsaicin alone and capsaicin plus kaempferol groups compared with the control group ( *p* < 0.001). In other words, capsaicin plus kaempferol reduced the abdominal writhing compared with the capsaicin group ( *p* < 0.001).

### Results of the tail-flick test

**Figure 2:**
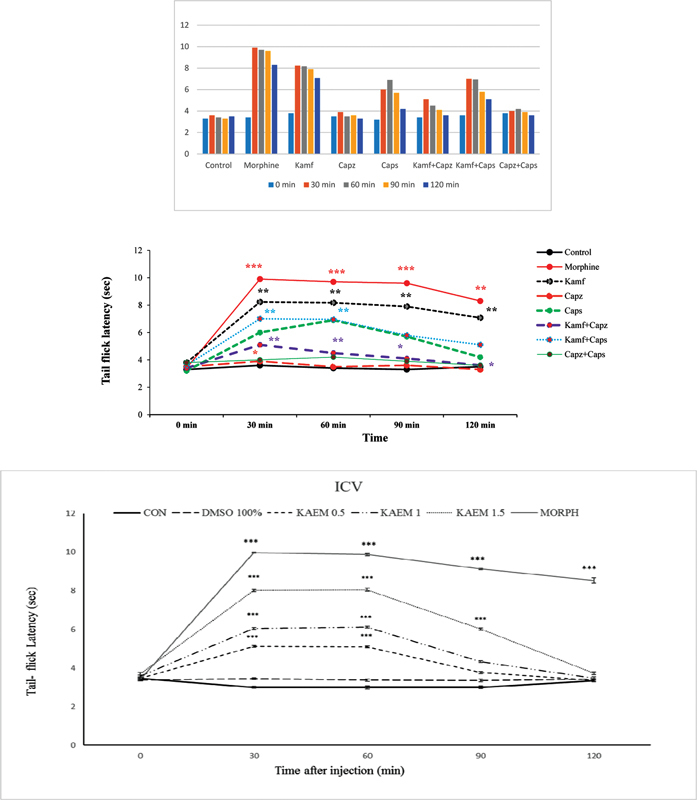
Comparison of the differences in reaction time in the tail-flick test. *** ( *p* < 0.001), ** ( *p* < 0.01), and * ( *p* < 0.05) compared with the control group; ### ( *p* < 0.001) compared with the morphine group; +++ ( *p* < 0.001) compared with the kaempferol group; !!! ( *p* < 0.001), !! ( *p* < 0.01), and ! ( *p* < 0.05) compared with the capsazepine group; ^^^ ( *p* < 0.001) compared with the capsaicin group. Abbreviations: KM, kaempferol; Capz, capsazepine; Caps, capsaicin.Each column represents the mean ± SEM.

According to Figure 2 , there was a significant difference between the experimental groups (F [7, 47] = 11.85, *p* < 0.001). Statistical analysis revealed that kaempferol led to a significant increase in tail-flick latency compared with the control group ( *p* < 0.01). In addition, morphine increased the tail-flick latency significantly compared with the other groups ( *p* < 0.001). Subsequently, the Tukey posthoc test showed that there was a significant difference between the capsaicin plus kaempferol and control groups ( *p* < 0.01). Capsaicin plus kaempferol caused significant analgesic effects and reduced pain compared with the control group ( *p* < 0.01). However, capsaicin alone caused no significant analgesic effects to reduce pain compared with the control group. Capsazepine administration alone did not reduce pain in rats compared with the control group.

### Results of the formalin test

**Figure 3:**
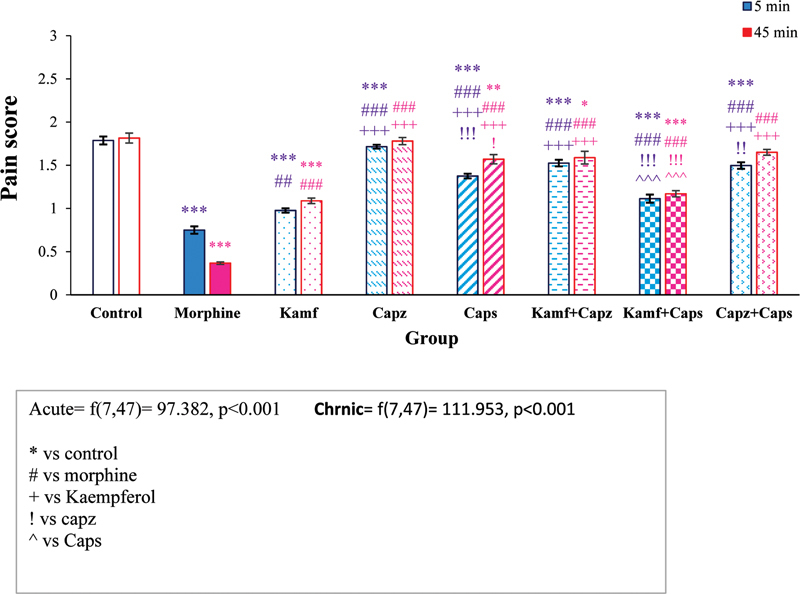
Comparison of the pain scores in phase 1 (nociceptive) and phase 2 (tonic) in the formalin test.

According to Figure 3 , there was a significant difference between the experimental groups in both phase 1 (5 minute nociceptive) and phase 2 (45 minute; tonic). In phase 1 of the formalin test, the experimental groups had significant differences (F [8, 53] = 122.163, *p* < 0.0001).

Administration of kaempferol significantly decreased pain scores in both phases compared with the control group ( *p* < 0.001). Also, morphine reduced pain scores in both phases compared with other groups ( *p* < 0.001). In the delay phase of the formalin test, injection of capsaicin alone could decrease the pain scores in comparison with the control group ( *p* < 0.001). In addition, capsaicin plus kaempferol led to a significant decrease in the pain scores compared with the control, capsazepine, and capsaicin groups ( *p* < 0.001).

In the late phase of the formalin test, the experimental groups showed significant differences (F [8, 53] = 178.652, *p* < 0.001). Kaempferol caused a significant decrease in pain scores compared with the control, capsazepine, and capsaicin groups ( *p* < 0.001). Also, the administration of capsaicin plus kaempferol caused a significant decrease in the pain score compared with the control, capsazepine, and capsaicin groups ( *p* < 0.001).

## DISCUSSION

In this research, kaempferol reduced pain in the tail-flick test. Kaempferol, as an herbal active constituent, could decrease pain possibly through TRPV1. The TRPV1 receptors are one of the effective systems involved in pain management that are expressed in different areas associated with pain in the central nervous system. 20 Moreover, kaempferol expression has been reported in some areas associated with pain in the central nervous system. 9 14 The standard abdominal writhing, tail-flick, and formalin tests have been used to investigate the anti-nociceptive effects of kaempferol and possible interaction with TRPV1 receptors. 21 22

The tail-flick test is usually used to evaluate the spinal reflexes and central analgesic pathways. The ICV administration of capsaicin plus kaempferol affected the central control of pain. However, capsazepine plus kaempferol had no analgesic affects. Capsazepine inhibited the effects of kaempferol in combination treatment. This finding is consistent with that of similar studies using the tail-flick test with an emphasis on TRPV1 receptors in the posterior periaqueductal gray (PAG) that is associated with pain relief. 23

In the abdominal writhing test, in which an intraperitoneal (IP) injection of acetic acid is used to evaluate peripheral antinociceptive activity along with inflammation, mediators, such as bradykinin, serotonin, histamine, substance P, and prostaglandin play an important role in inflammation. 26 All these mediators are associated with the stimulation of peripheral nociceptive neurons. 24 Based on our results, kaempferol prevented the abdominal constrictions induced by acetic acid, and it exerted its alleviative effects possibly through TRPV1 receptors. 13 The IP injection of acetic acid increases the amount of cyclooxygenase-2. Kaempferol suppresses cyclooxygenase-2 protein expression. 27 28 29 It can also inhibit inflammatory reactions associated with cyclooxygenase-2 expression. 27 30 Therefore, it can alleviate inflammatory pain. 30

Intracerebroventricular injection of capsaicin reduced the visceral pain effectively in comparison with the control group. It seems that capsaicin exerts its peripheral analgesic through at the level of central nerve endings. 13 In addition, the co-administration of capsaicin and kaempferol showed an analgesic effect. In other words, capsaicin using an intracellular mechanism increases the activity of TRPV1 receptors and prevents pain. 31 Co-administration of capsazepine and kaempferol caused significantly different effects than those of the kaempferol group. This means that capsazepine can block the kaempferol receptors, which inhibits the kaempferol effect; however, the underlying mechanism is not known. Nonetheless, the administration of capsazepine alone showed less analgesic effect.

The formalin test has been well established as a valid test for the screening of antiinflammatory and antinociceptive agents that act through the central nerves and neurons to assess peripheral pain. 32 33 The intraplantar injection of formalin evokes signs of nociception (licking, biting, and shaking of the injected paw) in the early phase, followed by a quiescent period, characterized by fewer pain behaviors, and at the late phase, which can last for ∼ 45 minutes. The early phase (nociceptive phase) results in the direct activation of peripheral nociceptors, 34 whereas the late phase (tonic phase) is due to increased production of prostaglandins and induction of cyclooxygenases and inflammatory nociception. 35 The late phase reflects the induction of a spinal state of facilitation, central sensitization, development of inflammation, and enlargement of receptive fields, as well as the concurrent presence of low-level input from both large and small afferents. 36

Our results showed that kaempferol had an inhibitory effect on the pain and showed antinociceptive activity in both phases of formalin-induced pain in rats. It was found that the analgesic effects of kaempferol were more potent in the late phase than in the early phase. Kaempferol facilitated the inhibition of the late phase of the formalin test, which seems to be an inflammatory response; thus, the antinociceptive effects of kaempferol are possibly mediated by the release of compounds, such as prostaglandins, which are partly sensitized by central nociceptive neurons. Therefore, kaempferol inhibited inflammatory mediators, such as cyclooxygenase-2. 37 Previous studies have shown that the inhibition of the N-methyl-D-aspartate receptor decreased intracellular calcium levels. 1 Consequently, the synthesizer enzyme of calcium-related nitric oxide and phospholipase A2 decreases, and due to a reduction in the levels of nitric oxide and prostaglandins, especially prostaglandins E2 and F2α, kaempferol exerts its antinociceptive effects. 38

The central injection of capsaicin in the late phase had a greater antinociceptive effect than in the early phase. Capsaicin may reduce pain by stimulating the descending antinociceptive pathways. 39 Capsazepine plus kaempferol caused results significantly different from those of kaempferol alone, and the analgesic effect of kaempferol was inhibited. Transient receptor potential cation channel subfamily V member 1 receptors play an important role in pain management. Flavonoids are known to have analgesic, anti-inflammatory, and antioxidant properties. These effects are related to the opioid and TRPV1. 40 In addition, intracerebral administration of TRPV1 activators induced analgesia, which is consistent with the results of the present study. 41 The activation of TRPV1 led to calcium influx. Also, depolarization of the neurons diminished action potential firing 42 and resulted in analgesia. 43

Activation of phospholipase C (PLC) led to the production of diacylglycerol (DAG). Capsaicin injection into the periaqueductal gray (PAG) area increased a delay in response to painful stimuli and glutamate release in the PAG area in rats, which was stopped by glutamate receptor antagonists. 44 Activation of TRPV1 by capsaicin through an increase in the number of DAG signals and the activation of the PLC pathway can lead to the release of glutamate from glutamatergic terminals, which is due to the joint activity of these two receptors. 45

According to the results of the present study, kaempferol plus capsaicin had a potentiating inhibitory effect on pain, and our results confirmed the ability of kaempferol to control pain. Studies have also shown that the central administration of kaempferol interacted with the endogenous system of pain control, which indicates the kaempferol effect on the central nerve endings. Our results are consistent with those reported in previous studies on similar flavonoids. 46

In conclusion, the TRPV1 antagonist blocked the analgesic effects of kaempferol, and TRPV1 receptors likely regulates the kaempferol analgesic effects. The kaempferol and TRPV1 channel are involved in the control of acute pain and can increase painful management. This study provides new information regarding the mechanism of antagonistic effects of kaempferol and the effect of a combination of capsaicin and kaempferol on the acute and chronic phases of pain (evidenced by the formalin test) in the central nervous system.
